# Supplementary material for: Patterns of Intron Gain and Loss in Fungi
Source: PLoS Biol. 2004 Nov 30;2(12):e422. doi: 10.1371/journal.pbio.0020422 (PMC532390; doi:10.1371/journal.pbio.0020422)
Supplement: Table S1 — Also available at http://genes.mit.edu/NielsenEtAl/. (4.3 MB ZIP). [file pbio.0020422.st001.zip › NielsenEtAl/html/358.html]

AN6915.1.NCU03714.1.MG08147.1.FG05144.1


```
 CLUSTAL W (1.82) Multiple Sequence Alignments - Introns Inserted


Sequence 1: NCU03714.1	573 aa
Sequence 2: MG08147.1	592 aa
Sequence 3: FG05144.1	575 aa
Sequence 4: AN6915.1	585 aa
Alignment Length: 609 aa
Number Identitical Residues: 164 aa
Alignment Score (without introns) 10389


MG08147.1 	MDVHLLIYDLSR~GMARQ~MSAGLLGFQLDAIYHTSIQLNGLEYVYDGGIVAIRPGSSHL
NCU03714.1	MDVHLLVYDLSR~GLAKQ~MSVGLLGFQLDAVYHTSIQLNGREYVYDGNIVSIIPGSSHL
FG05144.1 	MDVQLFVYDLSR~GLARQ~MSMGILGFQLDAIYHTSIELNGKEYVYDGGIIAIRPGSSHL
AN6915.1  	MDIELYVYDLSQ0GLARM0YSMALTGVQIDAIYHTSLVFNNTEYYFGQGIQTAQPSSTHH
          	**:.* :****: *:*:   * .: *.*:**:****: :*. ** :. .* :  *.*:* 

MG08147.1 	GQPLETQLLGQTHL~PMD--VVETYLDSMRSVYTAE~AYDLWKHNCNNFSNDFA~TFLLG
NCU03714.1	GRPLEEMYLGKTEF1QCADGIVPSANGSDRGVFRLT~EGNLHSR---------A1TFLLG
FG05144.1 	GQPLQRIPLGKTNL~PID--VIEEFLDSLRPIFTLE0AYDLFHHNCNNFSDSFV~NFLLG
AN6915.1  	GQPMEKIHIGTSEL~PLE--VVEEYLQSLSSIYTPE0SYDLFLHNCNNFTQDLA~MFLVG
          	*:*::   :* :.:      ::     *   ::      :*  :.... :.. .  **:*

MG08147.1 	HGIPDKILNMPSEVLESPLGRMLMPALDQQVRVNRQQNGGGMLGIGQNGSSGAAQHTSSQ
NCU03714.1	KGIPDYIVNMPQTVLSSPMGQMLMPMLNQQIHANKR--GGGILGIQQN-TPGSTSKPKAE
FG05144.1 	KGIPEHIVKMPQAVLDSPMGRMLLPQLTQGINAGRQ--GGSILGLQES--------SQAP
AN6915.1  	KSIPEHIRNLPTTFLSTPFGQMLKPQIESALRGVTQAPGQGLPAPRSG---PAGSHTQAQ
          	:.**: * ::*  .*.:*:*:** * : . :.   :  * .: .  ..    : . ..: 

MG08147.1 	ARASSHRVRQASNLRDLDSLMAQARSSCAVVFFTSATCPPCKMLYPMYEDLAEEVGDKGF
NCU03714.1	LHHHEGKVHNVTNLNELQSLLEKFQNSCAVVFFTSATCPPCKVLYPLYDQLAAEVGDKGV
FG05144.1 	AAAPKHVIKNIANASEFDRLMGEAKNSCAVVFFTSATCPPCKTLYPIYDELAEEVGDKAT
AN6915.1  	AQS-QGKVRVVGSLNELEQHLSSASKSCAVIFFTSATCPSCKMMYPVYDELAAEAGSKAV
          	    .  ::   .  :::  : .  .****:********.** :**:*::** *.*.*. 

MG08147.1 	VIKTDIS--VARDVAAKYGITATPTFITFLRGDQENRWMDADAGALRGNVRLLVQMAWPP
NCU03714.1	LIKVDVL--AAFDVGSAYSVSATPTFVTFLKGKQENRWSGADRGALQGNVRLLVQMAWPA
FG05144.1 	LMKVDISQPQQHEIGSRYSIRATPTIVTFLHGKEENRWSGADPAALRGNVQLLVQMAHPV
AN6915.1  	LLKVDTS--MAHDVAVRYGVHATPTFMTFLRGEKLDTWAGADAGKLRGNVRLLLEMAYPT
          	::*.*       ::.  *.: ****::***:*.: : * .** . *:***:**::** * 

MG08147.1 	HPHQSIRLPTIGKTEPNPVLFTKAPPLPKLTAKMGDKVAKDPAVESVIGFIMGRNSDGAA
NCU03714.1	HAHQSLNLPTFSNPGTKPVLYTKVPPLEKLLAKMG-SDAENPAVQGVKHFIEARSQDGPA
FG05144.1 	HPHERLRLPTFSNPNAKPVLYTKVPPLDKLMVKMGSEVASKPEVKALKQYLEDRTKNGPS
AN6915.1  	HPHRELRLPSFQRTITNYVTYNKTPPLEKLMQKLK-PHDQDPTLLAIIDFIKHRTTSSSA
          	*.*. :.**:: .. .: * :.*.*** **  *:     ..* : .:  ::  *. ...:

MG08147.1 	QAHLP-DVSAFSSFLESSIKTLEPEVLFPLVDVFRCALIDPRFSGCFAEEKDHKTIVTLL
NCU03714.1	EATLP-DMAAFTTFVRQSIKNVPTDTLFTVVDLMRIGLVDPRFSGYLAEEVVHGTVTALL
FG05144.1 	NAVIP-DMGHLSSLVKDSVASLPIDIFFTIVDLFRCALSDPRISGFFAEEKSHETVLAVL
AN6915.1  	DTPLPSDLPTFATYLAQTFHSTPQENRFALVDLVRVLFVDPRVAAYFGEDTSHTLLLTFF
          	:: :*:*:  ::: : .:. .   :  *.:**:.*  : ***.:. :.*:  *  : :.:

MG08147.1 	SHVNELPSCPYPLRLVTLQATANLFSSSLYWDPILR-HDGLRSQVIRLVSSSFLDEVHSS
NCU03714.1	EHINDLKECPYALRLVTLQMACNLFSSQLYADEVLS-NDKLRTCLTQLVSASFLDDNHNN
FG05144.1 	ESVAQKTDCPYALRLVTLQMACNFFSTPLFHDEIMR-DSSLRAAIILLVSSSFLDESHNN
AN6915.1  	STSEDEATS-YSLRIVTLQLACNLFKTPVYTNHLSSPSNPLRPALLSLVTSSLLN-AQSN
          	.   :   . *.**:**** :.*:*.: :: : :  . . **. :  **::*:*:  :..

MG08147.1 	VRVAAASVLFNVAVVNGRARRKGASAPGSTGINSPGDVLPDEDQVEVAASLVEAISQENE
NCU03714.1	VRVAAASLLFNIALANSRKRQ-----------DGPGDVLPQDDQIELAASILEAIAQEES
FG05144.1 	VRVAASSLLFNLSLANRKARS------------SSKPTLSGDDELELAASVVEAISLEEK
AN6915.1  	LRVVAASLAYNLAALNHNARFAN----------TGSEPLTEEEQVALTASVAEALAQESS
          	:**.*:*: :*::  * . *  .               *. :::: ::**: **:: *..

MG08147.1 	SKEGLEGMLLALGHLIYCLPLDGELADLIKTLDARRTVLGKKEDFKD--------MPLID
NCU03714.1	SGEALEGMLLALGYLAYRMPLDGELSDLLRTMDAGNSILEKKKAFPD--------MKLID
FG05144.1 	SAEALHGMLLALGHLVYGTSLDGELPDLLQAVGAQDSILAKKSKFPD--------EKLVT
AN6915.1  	STEALHGLLFSLGLLIYEVPMDGAVIDLCRALNLKEAVEGKKELITNGDQGKDKGDSLFR
          	* *.*.*:*::** * *  .:** : ** :::.   ::  **. : :........  *. 

MG08147.1 	EVGSVLLG-SLK--
NCU03714.1	EVGSELLGRGLNKP
FG05144.1 	EVGTELLGKGLRKP
AN6915.1  	EVGRELLAKGL---
          	***  **. .*
```
